# Participatory health councils and good governance: healthy democracy in Brazil?

**DOI:** 10.1186/s12939-015-0151-5

**Published:** 2015-02-19

**Authors:** Jillian Clare Kohler, Martha Gabriela Martinez

**Affiliations:** Leslie Dan Faculty of Pharmacy, Munk School of Global Affairs and Dalla Lana School of Public Health, University of Toronto, 144 College St, Toronto, ON M5S 3M2 Canada; Leslie Dan Faculty of Pharmacy, University of Toronto, 144 College St, Toronto, ON M5S 3M2 Canada

## Abstract

**Introduction:**

The Brazilian Government created Participatory Health Councils (PHCs) to allow citizen participation in the public health policy process. PHCs are advisory bodies that operate at all levels of government and that bring together different societal groups to monitor Brazil’s health system. Today they are present in 98% of Brazilian cities, demonstrating their popularity and thus their potential to help ensure that health policies are in line with citizen preferences. Despite their expansive reach, their real impact on health policies and health outcomes for citizens is uncertain. We thus ask the following question: Do PHCs offer meaningful opportunities for open participation and influence in the public health policy process?

**Methods:**

Thirty-eight semi-structured interviews with health council members were conducted. Data from these interviews were analyzed using a qualitative interpretive content analysis approach. A quantitative analysis of PHC data from the *Sistema de Acompanhamento dos Conselhos de Saude* (SIACS) database was also conducted to corroborate findings from the interviews.

**Results:**

We learned that PHCs fall short in many of the categories of good governance.

Government manipulation of the agenda and leadership of the PHCs, delays in the implementation of PHC decision making, a lack of training of council members on relevant technical issues, the largely narrow interests of council members, the lack of transparency and monitoring guidelines, a lack of government support, and a lack of inclusiveness are a few examples that highlight why PHCs are not as effective as they could be.

**Conclusions:**

Although PHCs are intended to be inclusive and participatory, in practice they seem to have little impact on the health policymaking process in Brazil. PHCs will only be able to fulfil their mandate when we see good governance largely present. This will require a rethinking of their governance structures, processes, membership, and oversight. If change is resisted, the PHCs will remain largely limited to a good idea in theory that is disappointing in practice.

## Introduction

The inclusion of citizens helps increase transparency and accountability in political decision making with regard to healthcare [1-3], as it provides civil society with information on the health system and health policies that can be used to monitor government practices [4]. Citizens can then raise concerns or challenge their local government and health authorities [5-9]. For instance, in Colombia, citizen involvement in the monitoring of funds allocated to key public sectors helped prevent as much as US$ 5.4 million from being lost to corruption [10]. In India, as another example, citizen participation helped expose that bribes were being demanded by physicians from mothers seeking treatment for their babies in maternity wards [2].

Historically, the Government of Brazil has aimed to include citizen preference in healthcare policymaking through advisory bodies such as Participatory Health Councils (PHCs). PHCs are “deliberative and permanent bodies of Brazil’s Unified Health System (*Sistema Único De Saúde*, or SUS), present in each sphere of the government (municipal, state, and national) and part of the basic structure of the Ministry of Health, the Secretary of Health of all states, and the Federal District of all municipalities” [11]. First created in 1937, they work as advisory bodies designed to monitor the healthcare system and foster empowerment among citizens [12-16]. PHCs have a seemingly ambitious mandate that includes setting guidelines for the preparation of health plans, monitoring the SUS, approving health projects, approving the proposed annual health budget, monitoring the allocation of health funds, forwarding any complaints, examining any irregularities, and monitoring the implementation of their own resolutions [11]. Today, they are present in 98% of Brazilian cities [17], demonstrating their popularity and thus their potential to help ensure that health policies are in line with citizen preferences.

Despite PHCs’ expansive reach, their real impact on health policies and health outcomes for citizens is uncertain [1,12,18,19]. The government has been subject to increasing citizen scrutiny and demands for better transparency and accountability of its decision-making processes. Citizen discontent, illustrated most recently by the Saúde +10 national movement that calls for the government to allocate 10% of Brazil’s Gross Domestic Product to health care [20], has put the government on notice that it needs to deliver health care services that meet citizens’ expectations. We thus pose the following questions: Do PHCs offer meaningful opportunities for open participation and citizen influence in the public health policy process? How does institutional architecture impact citizen participation in health policymaking?

Our paper is organized within the framework created based on the United Nations Economic and Social Commission for Asia and the Pacific’s (UNESCAP) definition of “good governance”, according to which good governance is “*participatory, consensus oriented, accountable, transparent, responsive, effective and efficient, equitable and inclusive and follows the rule of law. It assures that corruption is minimized, the views of minorities are taken into account and that the voices of the most vulnerable in society are heard in decision-making. It is also responsive to the present and future needs of society*” [21].

Considering each part of this definition in order, we outline our findings from our interviews with key informants from PHCs in Brazil at the municipal, state and national levels, and then triangulate them with data from the *Sistema de Acompanhamento dos Conselhos de Saude* (SIACS) database. Where the clauses of the definition contain subcomponents, we break down our discussion into sections devoted to each subcomponent.

## Methods

### Data collection

#### Key informant interviews

Thirty-eight semi-structured in-depth interviews were conducted with health council members using purposive sampling. Eight municipal, seven state and six national health council members were interviewed in Portuguese over the phone in June and July of 2013. An additional nine municipal, seven state, and one national health council members were interviewed in May of 2014, totalling 17 municipal, 14 state, and 7 national health council member interviews. General questions regarding their respective PHCs such as council structure, council activities, and everyday operations were asked during the interviews. Study participants were identified by accessing PHC membership lists that were available online in various PHC websites at the national, municipal, and state levels. We contacted all council members in the available membership lists by sending an email invitation letter to participate in the study. This resulted in about 1,400 email invitations. Non-respondents were contacted on two separate occasions via email and telephone (when a phone number was available) to solicit a response. We also used snowball sampling. Interviews were conducted in Portuguese until theme saturation became present.

#### SIACS database

The *Sistema de Acompanhamento dos Conselhos de Saude* (SIACS) online database was used in May of 2014 to extract the following data about all PHCs registered in the database: the number of council members that are “users”, “health professionals”, “private sector”, and “government representatives” in each PHC; the total number of council members; whether elections were held for the PHC presidency; whether the PHC has its own headquarters; whether it is in control of its own budget; whether it conducts training for council members; and whether it lacks internet access. When the data were retrieved, only 4,113 of the total 5,570 municipalities had fully completed their registration on the database. The 4,113 Municipal Health Councils (MHCs) with data available, as well as the 27 state councils and the National Health Council (NHC), were part of this analysis.

### Data analysis

#### Key informant interviews

Interviews were transcribed and then translated into English. Qualitative interpretive content analysis was used to analyze the interview data. It is a research method used to examine meanings, themes and patterns in text, facilitating the understanding of social reality in a subjective but scientific manner [22,23]. Data were systematically classified by coding and identifying themes. To make valid inferences from the data, we ensured that our categorization process was consistent and that the three independent researchers who coded the data did so following the same procedures. If coding discrepancies were found, they were discussed until consensus was reached. Coding stages were iterated until all categories were identified and links established and facilitated by using the components of the UNESCAP definition of good governance to organize our themes, as presented below. The software HyperRESEARCH was used to help manage and organize the data.

#### SIACS database

We triangulated our key informant interviews with data from the SIACS database. The retrieved data were used to calculate the average number of PHCs that: 1) hold elections to select the PHC president; 2) follow the parity principle; 3) have their own headquarters; 4) have control over their budget; 5) train their council members; and 6) have Internet access. The percent average of each category was calculated using a simple average formula and rounded up to the nearest integer:

$$ \mathrm{Percent}\ \mathrm{average} = \left[\left({\mathrm{a}}_1 + {\mathrm{a}}_2 + {\mathrm{a}}_3,+...... + {\mathrm{a}}_{\mathrm{n}}\right)/\mathrm{n}\right] \times 100 $$

### Ethical considerations

Our research was approved by the University of Toronto’s Research Ethics Board and Brazil’s National Research Ethics Commission (*Comissão Nacional de Ética em Pesquisa* (CONEP)). This study was funded through an operating grant from the Canadian Institute of Health and Research (CIHR).

## Results

### Participatory

#### Training and informed participation

According to UNESCAP, “participation could be either direct or through legitimate intermediate institutions or representatives. Participation needs to be informed and organized. This means freedom of association and expression on the one hand and an organized civil society on the other hand” [21].

A crucial challenge to informed participation in PHCs is a reported lack of training of council members at all levels of government (Table 1). This contradicts the government’s National Policy for Permanent Education (2006), which guarantees budget allocation for PHCs at all three levels of government for the training of council members [24]. A SHC member stated,

**Table 1:** Results from in-depth interviews*

	**Observed results**
**Participatory**	• Lack of training of council members
	• “Politics” influences discussion topics
	• Tense relationships between council members at the national level
	• Lack of organized civil society groups to participate in PHCs
**Consensus oriented**	• The parity principle is not enforced (*excluding the national level*)
	• Weak guidelines for mediating different interests
	• Predominant individualistic interests
**Accountable**	• No way to measure PHC outcomes, making it difficult to measure accountability to the public
**Transparent**	• Delays in implementation of deliberations due to a highly bureaucratic system that lacks transparency and lack of government support
	• Information is disseminated via newspaper, as per the law
	• Need to use other modes of information dissemination to address lack of public knowledge about PHCs
**Responsive**	• Lack of government responsiveness due to bureaucratic system and inadequate government support
	• PHCs are responsive to societal movements but have a limited impact on fulfilling their demands
**Effective and efficient**	• PHCs provide a forum for discussion of health issues, but real impact remains inconclusive
	• Inefficient budget allocation process (*excluding the national level*)
	• Budget constraints cause a lack of resources (*excluding the national level*)
**Equitable and inclusive**	• Cordial relationships between council members (*excluding the national level*), with a lack of interaction between them
	• Limited inclusiveness due to membership guidelines
**Follows the rule of law**	• PHCs do not seem to follow their mandate with matters related to the SIACS database, the parity principle, and training of council members
	• PHCs lack an impartial body to enforce their legal framework
	• PHCs are vulnerable to corrupt practices

*All findings are consistent with PHCs at the national, state, and municipal levels, unless otherwise indicated.

[*It is apparent*] *that user representatives are limited by their education levels and knowledge. The training provided is definitely not enough.*

Almost all key informants reported a lack of council member training. A council member at the municipal level stated,

*There is no support for training from the* [*municipal*] *government despite constant requests. Everything that is done in the Council is done by us.*

The SIACS database revealed that only 59% of municipal health councils (MHCs) and 85% of state health councils (SHCs) provide training for their council members, and that there is no training at all at the national level (Table 2). Given the technical nature of many health system issues, some understanding of core concepts is vital for constructive dialogue to take place. Asymmetry in health system knowledge gives those that have advanced education or a greater general knowledge of the health system an advantage in understanding and voicing their demands and concerns [5], creating a power imbalance with those who do not have such technical knowledge [25]. This knowledge gap can have a silencing effect on council members who are not well informed as a result of improper or insufficient training. Even when they occur, training efforts are limited by a high turnover rate caused by a lack of attendance, new leaders and new associations [26,27].

**Table 2:** **Results from the SIACS database**

	**Hold presidential elections**	**Follow parity principle**	**Have headquarters**	**Control their budget**	**Provide training to their council members**	**Lack access to internet**
National level (n = 1)	100%	100%	100%	100%	0%	0%
State level (n = 27)	59%	81%	52%	78%	85%	4%
Municipal level (n = 4113)	88%	73%	29%	33%	59%	21%

#### The role of politics

The PHC leadership is composed of a council president and a board of directors, both of which are elected by council members. They are in charge of setting the agenda, distributing meeting materials within and outside PHCs and guiding discussions. Key informants often pointed out that “politics” influences what issues PHCs focus on during discussions as well as the relationships among council members (Table 1). A council member at the municipal level stated,

*The government tries to dominate PHCs in a very subtle way, like by including people who are close to them to advance their agenda.*

Tension often exists between user representatives, government representatives, and health professionals at the national level (Table 1), perhaps as a result of individualistic interests. At the state and municipal levels, this does not seem to be an issue, as key informants described relationships among council members as rather cordial and respectful.

#### Participation by Organized Civil Society

At all three levels of government, key informants reported that members of civil society do not actively participate in PHCs because they are not organized and are often not aware that they can play a role in health policy through such participation (Table 1). A municipal-level PHC member pointed out,

*Brazilian society has to evolve to overcome the present lack of organization and interest in order to engage in the development of better health policies.*

Another member at the state level noted,

*I would like to see civil society participate in PHCs the way it did in the 70s and 80s through the Diretas Jà Popular Movement, going on the streets and asking for our rights. That guaranteed the inclusion of SUS in our current Constitution (1988). Nowadays people are too comfortable to participate.*

### Consensus oriented

#### The parity principle

According to UNESCAP, “good governance requires mediation of the different interests in society to reach a broad consensus on what is in the best interest of the whole community” [21]. In their formal processes, PHCs aim to mediate various societal interests through guidelines such as the parity principle, which legislates that the number of user representatives must be equal to that of health representatives and government representatives combined [12]. In practice, the parity principle is not enforced in PHCs at all three levels of government (Table 1). A council member at the municipal level stated,

*I have always defended SUS and the PHCs. However, when I started to participate in PHC meetings, I could see that civil society is not well represented. The current model is failing.*

The SIACS database indicates that the NHC has adopted the parity principle, but only 81% of SHCs and 73% of MHCs enforce it (Table 2).

#### Other guidelines to ensure mediation of various interests

There are other guidelines in place to mediate different societal interests, such as limiting the amount of time a person can speak during PHC meetings and complying with the process for adding items to the discussion agenda. Still, many council members expressed that PHC meetings are unproductive due to long and repetitive discussions, particularly at the state level (Table 1):

*…new mechanisms for discussion would help meetings be more focused. They are repetitive, exhausting, with long speeches that lack focus.*

These reports suggest that the current guidelines are ineffective and need to be revised to ensure that PHCs are consensus oriented. This is particularly important given the reported high level of narrow interests exhibited by council members (Table 1). A council member at the national level stated,

*The interests of each individual group predominate over the collective goal of PHCs. Selfishness can be perceived very easily.*

Council members may abstain from engaging in discussions that do not directly involve their constituents’ agenda, and may often disregard resolutions that are advantageous to society in general but not specifically to the entities they represent.

#### Motivations for PHC membership

Council members’ motivations for participating in PHCs differed widely; some at the municipal level participate in PHCs for personal reasons such as to learn about the health system. At the national level, many said that they participate because it is strategic to do so, as PHCs provide an opportunity to sway discussion and government resources to benefit their own interests. A national-level key informant said,

*Participating in PHCs is a strategic action that allows my entity to have a voice throughout the year, not just during elections.*

Council members’ constituents’ agenda takes a leading role in the discussions that take place in PHCs as well as the deliberations that are put in place. Council members may also view their participation as a pathway for better opportunities [26]. Membership is therefore not only grounded on altruism, as personal gains also come into play in membership motives. A state-level key informant noted:

*Some user representatives start participating in PHCs without even knowing what a health council is or what it does. Can you believe it?*

### Accountable

UNESCAP states that “accountability is a key requirement of good governance. Governmental institutions as well as the private sector and civil society organizations must be held accountable to the public and to their stakeholders. “In general, an organization or an institution is accountable to those who will be affected by its decisions or actions” [21]. There is an absence of guidelines to monitor PHC deliberations or to take stock of their accomplishments. In light of this, while we question how accountable the PHCs are to their constituents, it is difficult to effectively assess their level of accountability to the Brazilian public.

### Transparent

#### The role of PHCs

UNESCAP explains transparency as follows: “Transparency means that decisions taken and their enforcement are done in a manner that follows rules and regulations. It also means that information is freely available and directly accessible to those who will be affected by such decisions and their enforcement. It also means that enough information is provided and that it is provided in easily understandable forms and media” [21]. Increasing transparency in decision making by including civil society in the formulation of policies is one of the main objects behind the creation of PHCs [28]. PHCs have pushed governments to report health-related activities, particularly in relation to the allocation of health budgets. The SIACS database has also increased transparency by making information on PHCs publicly available online. To be sure, it is unclear how extensively this database is used and updated.

#### Adherence to rules and regulations

Council members reported delays in the implementation of PHCs’ decisions at all three levels of government. PHCs rely solely on their respective governments for policy implementation.

Ensuring that PHC deliberations are implemented is limited by a reported lack of transparency caused by a highly bureaucratic system (Table 1). This coupled with a reported lack of government commitment to implement PHC decisions results in a political environment that lacks transparency and makes it difficult to ensure that rules and regulations are being followed in order to implement PHC decisions.

#### Public access to information

PHCs are required to publish the resolutions, recommendations, and proposals that take place at PHC meetings in their respective official local newspapers within 30 days. The Brazilian government has also implemented the SIACS database to disseminate information on PHCs and to increase transparency and access to information to the general public via the Internet. PHCs are obligated to provide information on the number of council members, entities represented, agenda topics, resources, and mandate on SIACS. However, as of May 20, 2014, only 73.8% of Brazil’s PHCs had uploaded such information to the database.

Council members at all three government levels reported that information is disseminated in newspapers as well as through the Internet or on the radio. While the NHC has an extensive amount of information available to the public on its website, some SHCs and various MHCs have limited to no information available online. This could be a result of a lack of Internet access by PHCs, particularly those at the municipal level and in remote areas. As many as 21% of MHCs lack access to the Internet (Table 2). Even if the PHCs intensify how they use the Internet to disseminate information, about 60% of Brazilian households, particularly in the northern states and remote areas that have limited telecommunication networks [29], lack Internet access [30].

### Responsive

#### Institutional Responsiveness to PHCs

Responsiveness as defined by UNESCAP “requires that institutions and processes try to serve all stakeholders within a reasonable timeframe” [21]. Key informants reported that PHC resolutions are often not implemented or take a long time to go into effect due to a lack of government support and a bureaucratic system (Table 1):

*Council members constantly push the government to implement the decisions taken in the council without much success.*

Another council member at the municipal level stated,

*There is a huge gap between state and municipal PHCs. The Health Council of my state does not support us at all. What about the NHC? That is the worst one. They do not even know that my municipality exists.*

Many of the council members interviewed expressed general frustration due to their unsuccessful attempts at encouraging their local governments to implement decisions taken during PHC meetings. This directly limits the capacity of PHCs to be responsive to the public.

#### PHC responsiveness to the Brazilian population

Council members at the municipal and state levels reported that PHCs tend to be responsive to demands from social movements. Key informants reported that PHCs assisted in the support of the Saúde +10 movement. Key informants at both levels reported having discussions during PHC meetings about this movement, and one interviewee at the state level reported that signatures were collected by his PHC to support it. However, at the national level, one interviewee reported that while such support forced the government to think about this issue, it failed to have any meaningful long-term impact, stating that “[*the support*] *ended as fast as it began*”. At the municipal level, a council member also reported,

*I hope that one day PHCs change in order to listen to society. The system we have today doesn’t at all; we just pretend to listen.*

These findings suggest that PHCs have the ability to be responsive to societal needs and demands, but that they are undercut by the fact that they lack the necessary power and support (Table 1).

### Effective and efficient

#### PHCs’ effectiveness in health policy

Based on UNESCAP’s definition of good governance, “processes and institutions” are effective and efficient when they “produce results that meet the needs of society while making the best use of resources at their disposal” [21]. Accordingly, we conducted an analysis of PHCs’ level of perceived effectiveness from key informants as well as the level of efficiency in the allocation of PHCs’ budgets.

Council members at all three levels of government generally reported that PHCs are effective insofar as they create a forum through which different societal groups can come together to discuss health issues and exercise participatory governance. A council member at the municipal level stated,

*by participating in PHCs, civil society is able to monitor health policies and manage health issues* [*one*] *is interested in.*

While this seems like a positive outcome, it is difficult to measure PHCs’ actual impact on health policies (Table 1), making it uncertain whether they have the ability to produce policies that will benefit society at large.

#### PHCs’ use of resources

Council members at the national and municipal levels noted that there are issues in how the PHC budget is allocated. Four council members at the municipal level and two at the state level felt that the budget was not used effectively, while many others reported that there is a lack of transparency in the way in which the budget is spent (Table 1). The budget allocation process was described as lengthy, bureaucratic, and difficult, as it is often the Health Secretariat that is solely in charge of deciding how the budget is spent. A council member at the state level reported,

*It is obvious that politics is involved in the approval of how the budget is spent within the council. We have our own budget, but it’s almost impossible to get approval to use it.*

Council members at the municipal level also reported a lack of resources caused by budget constraints, which limits PHCs’ ability to fulfil their mandate. At the national level we did not have any reported budget allocation issues.

### Equitable and inclusive

UNESCAP defines an “equitable and inclusive” organization as follows: “… all [council] members feel that they have a stake in it and do not feel excluded from the mainstream of society. This requires [that] all groups, but particularly the most vulnerable, have opportunities to improve or maintain their wellbeing” [21]. Using this definition, we focused on exploring the relationship between council members as well as the opportunities PHCs bring to civil society groups.

#### Relations among council members

Council members reported that relationships between members are generally respectful and positive, particularly at the municipal level (Table 1). At the state level, relationships were also described as positive, with the only complaint being a lack of interaction between users, health professionals, and government representatives. At the national level, council members reported tensions between users and government representatives.

#### Inclusivity

The PHC mandate requires that civil society groups with PHC membership must be organized and be active in at least one-third of all Brazilian states and in at least three of the five geographical regions of the country [31]. As a result, civil society membership often reflects a pre-existing network of relationships between organized civil society groups and the government, excluding groups that lack such government ties and organization, which may also be the most marginalized, from decision-making (Table 1) [1].

*Most of the studies about PHCs say that the health councils are important because they represent society. That is not true. PHCs are not what we all dreamed of.*

Many key informants stressed the importance of raising awareness about PHCs in order to engage the most marginalized groups in society in health policy formulation and discussion. This will ideally help make PHCs more equitable and inclusive, and will also push for greater accountability for council deliberations from the government; by default, this will help PHCs have a greater impact on health policies.

### Follows the rule of law

#### PHCs’ failure to follow policies

UNESCAP explains that an organization follows the rule of law when “fair legal frameworks are enforced impartially. Impartial enforcement of laws requires an independent judiciary and an impartial and incorruptible police force” [21]. Perhaps because the judiciary and police play no part in enforcing behaviour by the PHCs, they frequently fail to abide by binding principles:

- Council members reported that PHCs often do not adhere to the legal guidelines that were updated and redefined in 2012 for everyday council activities [17], particularly in relation to data banks, membership composition, elections and the training of council members.
- As mentioned, more than a quarter of PHCs have failed in their obligation to upload and update PHC information (council composition, infrastructure, founding legislations, agenda topics etc.) to the SIACS database.
- The SIACS data analysis showed that only 81% of SHCs and 73% of MHCs follow the parity principle (Table 2), even though it is mandatory and legislated under PHC mandate.
- PHC presidents must be elected by secret ballot by council members, for a one-year term with the possibility of re-election [31], but data analysis from SIACS has shown that 41% of SHCs and 12% of MHCs do not hold elections for PHC president (Table 2). This suggests that the PHC presidents may likely be political appointees that will then influence the agenda of the PHCs contrary to the principle of rule of law.
- Last, we found that despite a government policy (the National Policy for Permanent Education) that legislates the government’s responsibility to provide budgets for the training of PHC council members, there is little evidence of this taking place.

#### Absence of an Independent body to enforce PHCs’ legal framework

Due to the advisory nature of PHCs’ mandate, they rely solely on governmental support for the implementation and enforcement of their legal framework both within and outside PHCs (Table 1).

This is problematic, as council members reported that there is a lack of government support, recognition and respect for PHCs. A council member at the municipal level stated,

*Having a council is obligatory. For this reason the government supports their existence but not what they were made to do.*

At the national level, key informants reported that any form of government support often comes with strings attached. These findings correlate with the existing literature, which highlights that while participatory democracy is a new mode of governance, it can increase vulnerabilities for government manipulation in the decision-making process [32,33]. Political influence in the form of “favour exchanging” often takes place, as council members use PHCs to advance personal gains by promoting the government’s agenda (Table 1):

*PHCs are like any other forum for political discussions in the country. There are favour exchanges, corruption, and everything in between.*

## Discussion

By using UNESCAP’s definition of good governance as our organizational framework, we discovered that PHCs have substantial weaknesses. PHCs have failed to effectively reach out to civil society’s most marginalized groups, which puts into question their accountability and inclusiveness. There is a need for stronger guidelines to ensure that PHCs are transparent in their policies and processes. In addition, more effective information dissemination to Brazilian society at large would help increase accountability. We also found a need for deeper training and education of PHC council members. Unless council members are knowledgeable about health system issues, discussions and deliberations within the PHC will remain superficial. These findings are an important addition to the existing international literature, as they illustrate that civil society participation alone may not be a viable tool for increasing accountability and improving service delivery in the health system.

Our research has some limitations. First, there is wide acceptance of the fact that a lack of good governance can create barriers to development. While the cultural and systematic implications of using UNESCAP’s definition of good governance go beyond the scope of this paper, we want to point out that “good governance” and its corresponding definition are based on democratic principles [34]. The UNESCAP definition and indicators of good governance thus may not be fully applicable in other country contexts due to differences in socioeconomic, political, cultural, legislative, and historical factors. In addition, we recognize that researcher biases such as ideology, beliefs, and the like may have influenced the review process and that the choice of using a traditional literature review method may result in unreported findings. While we only interviewed 38 council members out of the 54,306^a^ that exist throughout Brazil, our data reached saturation. Moreover, key informants who agreed to participate in our study may have added an inherent bias to our data, as they may have projected their ulterior motives onto the answers collected during the interview process. And given that our interview participants were recruited through email and our interviews were conducted over the phone, we may not have been able to reach out to marginalized populations who lack access to such modes of communication. Because interviews were done over the phone, our findings may be limited by a reduction of social cues. Lastly, personal biases may have influenced our findings during the interview transcription stage of our data analysis.

## Conclusion

Our research was directed towards exploring whether PHCs offer authentic opportunities for open participation and influence in the public health policy process. What we found is that even though PHCs are intended to be inclusive and participatory, in practice they seem to have little impact on the health policymaking process in Brazil. We learned that PHC deliberations are often narrow and controlled. Consequently, we did find that a stronger level of trust is needed between all three council member groups to ensure that political affiliations or work contracts do not impede freedom of association and prevent free expression from taking place [26]. The limited involvement of civil society also hinders how well societal interests are represented within the PHCs. Without repeating all of our findings, we found that the PHCs fall short in many of the categories of good governance, including government manipulation of the agenda and leadership of the PHCs, delays in the implementation of PHC decision making, a lack of training of council members on relevant technical issues, the largely narrow interests of council members, the lack of transparency and monitoring guidelines and a lack of inclusiveness. PHCs will only be able to fulfil their mandate when we see good governance largely present. This will require a rethinking of their governance structures, processes, membership and oversight. If change is resisted, the PHCs will remain largely limited to a good idea in theory that is disappointing in practice.

## Endnote

^a^This number is based on the number of Participatory Health Councils registered on the SIACS database as of December of 2014 (73.27% of the total number of PHCs in the country).

## Abbreviations

MHC: Municipal Health Council
NHC: National Health Council
PHC: Participatory Health Council
SHC: State Health Council
SIACS: Sistema de Acompanhamento dos Conselhos de Saude
SUS: Sistema Único De Saúde
UNESCAP: United Nations Economic and Social Commission for Asia and the Pacific
